# Microbial Consortium–Mediated Degradation of Polyethylene Terephthalate in Orthodontic Aligners: A Comprehensive Review

**DOI:** 10.1155/ijm/7509196

**Published:** 2025-09-15

**Authors:** Sivakamavalli Jeyachandran, Mohammed Aman

**Affiliations:** ^1^Lab in Biotechnology and Biosignal Transduction, Department of Orthodontics, Saveetha Dental College and Hospitals, Saveetha Institute of Medical and Technical Sciences (SIMATS), Saveetha University, Chennai, Tamil Nadu, India; ^2^Department of Industrial Engineering, College of Engineering, University of Business and Technology, Jeddah, Saudi Arabia

**Keywords:** biodegradation, biomedical plastic waste, microbial consortia, orthodontic aligners, PETase enzyme, polyethylene terephthalate

## Abstract

In this review, we critically assess microbial consortia–mediated biodegradation of polyethylene terephthalate (PET) used in orthodontic aligners as a means to address the environmental problems arising from biomedical plastic waste. PET's chemical stability and high crystallinity provide sufficient durability for clinical efficacy; however, natural degradation is hindered in the resulting environmental accumulation. Currently, the conventional disposal methods can lead to toxic emissions, so they have to be kept in the dark in terms of innovations that reduce or eliminate them. These microbial consortia utilize hairy dynamics of synergistic enzymatic activities, that is, PETase and MHETase, to degrade PET into assimilable monomers, with the potential to be a bioremediation strategy. In this paper, we summarize current analytical methodologies to assess degradation, present molecular tools to elucidate microbial community dynamics, and discuss biotechnological strategies to enhance enzymatic efficiency and process scale-up potential. Slow degradation rates, material complexity, and environmental variability are identified as challenges. Advances in enzyme engineering, bioaugmentation, and bioreactor design associated with biodegradation performance are reviewed, and future research directions that will enhance the ecosystemic performance of microbial consortia for eco-friendly and effective management of PET waste from orthodontic aligners within the framework of a circular bioeconomy are pointed out.

## 1. Introduction

Polyethylene terephthalate (PET) is one of the most widely used synthetic thermoplastic polymers in industries owing to its prominent physical and chemical properties. PET has been established as a preferred material for the fabrication of orthodontic aligners; orthodontically speaking, these are transparent dental devices that have the duty of gradually correcting malocclusion [1], due to their durability, optical clarity, chemical inertness, and biocompatibility. The key modification that changed orthodontic practice is the introduction of clear aligners, as the aesthetics, comfort, and removability of this metallic brace alternative have significantly increased patient compliance and quality of life while wearing them [2]. Although the clinical advantages of PET-based aligners have been proven, the increase in environmental concerns for the usage of PET-based aligners has made it difficult to take this orthodontic appliance to market. However, due to the short useful lifespan of orthodontic aligners (made to be replaced a few weeks apart) and their large-scale disposal, these biomedical plastics contribute to the global burden of plastic waste, produced annually [3]. A study reports that the aromatic band at 1616 cm^−1^ in PET's chemical structure, consisting of a highly stable aromatic polyester backbone, is resistant to natural degradation processes such as hydrolysis and photodegradation and, consequently, is persistent in terrestrial and aquatic environments for a prolonged period [4]. PET waste is accumulating, contributing to microplastic pollution that comes with bioaccumulation and trophic transfer [5].

Currently, the disposal of PET biomedical waste is by incineration and landfill deposition. Nevertheless, incineration can be a source of emission of harmful substances, principally dioxins and furans, that threaten air quality and public health [6]. Conversely, landfilling is long-term contamination of soil and groundwater resources via leaching of additives and degradation products to the site [7]. Given this, there is an immediate necessity for creating sustainable and functional waste management strategies for PET incorporating biomedical materials, including orthodontic aligners, in line with wider aims for a circular economy and responsible environmental practices in healthcare [8] and bioremediation of PET waste through microbial consortia–mediated biodegradation has emerged as a viable pathway. The integrated effects of microbial diversity and interspecies interactions, which distinguish consortia from single microbes, include a more extensive enzymatic repertoire and greater resilience to environmental changes [9]. Cooperation in this way can help promote the decomposition of complex polymers via sequential or parallel enzymatic reactions until the polymers are ultimately mineralized into environmentally friendly substances [10]. Microbial PET degradation pathways have been elucidated, with some of the greatest progress occurring following the discovery of *Ideonella sakaiensis*–driven production of catalytic enzymes PETase and MHETase, which hydrolyze PET back to its monomers, terephthalic acid (TPA) and ethylene glycol (EG) [11]. Studies that followed have looked at optimizing microbial consortia with the species that produce PETase, improving the stability of the enzyme itself and engineering microbes for better PET degradation efficiency [12]. Such advances have specific pertinence to the PET waste produced from biomedical applications, where conventional recycling paths are constrained by contamination and mixed material feedstock [13]. In this review, current research on microbial consortium–mediated PET degradation for biomedical plastics such as orthodontic aligners is critically reviewed (Table 1). This review seeks to define enzymatic and microbial mechanisms, determine challenges that are both process scale and environmental constraints, and discuss novel approaches to biotechnological innovation toward sustainable use and management of dental material waste.

### 1.1. Background on PET Usage in Orthodontic Aligners

**Figure 1:**
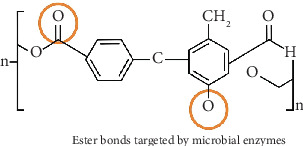
Structure and composition of polyethylene terephthalate (PET).

PET is a semicrystalline thermoplastic polyester with a wide range of utility in a number of biomedical and industrial applications. The molecular architecture of this polymer is its repeating ester linkage between TPA and EG, producing a polymer chain having a highly stable aromatic backbone (Figure 1). PET exhibits considerable mechanical strength, chemical resistance, and thermal stability as a result of this structural configuration, enabling the material for applications that demand high performance like food packaging, textiles, and medical devices [20]. Proven to be biocompatible and possessing suitable physical properties, PET has been established for use in the fabrication of orthodontic aligners, in which material transparency, flexibility, and durability are important. Materials that can withstand the biomechanical forces involved in moving teeth while providing a deceivingly long wearing period and do not dent or diminish in aesthetics are necessary in orthodontic aligner production. These criteria are fulfilled by PET, which conforms to the manufacture of optical devices and provides a clear, glass-like transparency resulting in the near invisibility of the aligners during use and helps in patient acceptance [21]. Additionally, its semicrystalline nature makes PET flexible, which allows aligners to adapt exactly to particular dental arches, providing the necessary controlled orthodontic forces for successful tooth repositioning.

Rapid uptake in demand for clear aligner therapy over the past decade was a result of technology and consumer preference shifts toward less-invasive, more cosmetically favorable orthodontic options. On the one hand, traditional fixed metal braces are effective but have an aesthetic drawback; meanwhile, they constitute problems when it comes to oral hygiene, something that clear aligners can handle. Aligners made from PET represent a removable, comfortable, and discreet option for a wide demographic range of patients, including teenage and adult patients that look for orthodontic correction but without the perception or discomfort of the conventional appliances [22]. The usage of PET aligners with dental care has globally appeared clinically, in accordance with the development of digital dentistry, 3D printing technology, and computer-aided design/manufacturing (CAD/CAM) technology, which helps precise customization of Invisalign by using patient-specific dental scans [23]. Although PET has favorable clinical and material properties, the posttreatment waste management issues associated with PET will be discussed. Unfortunately, PET's biodegradation-resistant chemical robustness—the backbone to its performance—is not prone to degradation by microbes. Under natural environmental conditions, its aromatic polyester backbone makes this synthetic recalcitrant to hydrolytic and enzymatic breakdown, resulting in long persistence in waste streams [24]. As orthodontic treatment protocols generally tend to last several months to years, patients are required to replace aligners every 1–2 weeks. Due to repeated disposal, cascading effects of some large quantities of biomedical plastic waste (mainly PET) are generated in both clinical and domestic settings [25]. The accumulation of such polymeric waste has environmental connotations that require the generation of innovative recycling and disposal methodologies that are appropriate specifically for the special PET application in orthodontic applications. Solving these problems is fundamental to protect the environmental sustainability of the benefits of advanced orthodontic treatment.

### 1.2. Environmental Concerns of PET Accumulation and Plastic Waste From Dental Appliances

PET use in orthodontic aligners has proliferated to the point where environmental footprints have grown alarmingly large, and there are fears about the long-term ecological legacy from discarded dental plastics. PET has a density of 1.35 g cm^3^ and is structurally inert, with a high aromatic ester containing a plurality of aromatic ester linkages that impede various degradation mechanisms, including photolytic, hydrolytic, and microbial processes [26]. Therefore, PET waste generated by orthodontic applications typically lingers in the environment for decades or even longer, in landfill sites or discharged through inappropriate disposal pathways into aquatic systems. This persistence contributes to the global crisis of plastic pollution in which microplastic contamination is an emergent environmental threat; that is, the plastic particles are smaller than 5 mm and widely spread over the environment [27]. In the dental sector, clinics and orthodontic practices create large amounts of PET waste from discarded aligners, quantities of packaging materials, and related biomedical plastics. Most of the latter are disposed of through conventional methods such as landfilling and incineration, which are environmentally dangerous. Landfilling PET waste results in long-term environmental persistence and has the capability for additive leaching and microplastic formation through physical fragmentation processes. These microplastics may then be transported into soil and groundwater systems and become a threat to terrestrial organisms and may lead to human water supply contamination [28]. In the reduction of throughput but not in the toxic release of dioxins, furans, and polycyclic aromatic hydrocarbons which contribute to air pollution and elevated greenhouse gas emissions exacerbating climate change [29]. The disposal costs faced by these routes underline the deficiency of the present waste management for the biomedical PET plastics. The ecological and human health concerns associated with microplastics that derive from the fragmentation of discarded PET aligners are of particular interest. Small size, high surface area-to-volume ratio, and high mobility make microplastics facile vehicles of environmental contaminants like heavy metals, persistent organic pollutants, and microbial pathogens [30]. Adsorption of toxic substances to sediment facilitates their bioaccumulation in aquatic and terrestrial food webs, with flow through organisms at higher trophic levels, such as humans, while life experiment results have revealed that ingestion and inhalation of microplastics can lead to adverse biological effects such as inflammation, oxidative stress, and disturbance of endocrine function [31]. In addition, the presence of microplastics can inhibit feeding, subsequent reproduction, and growth among many wildlife species, thereby affecting biodiversity and ecosystem resilience [32]. With such multidimensional impacts, the environmental implications of PET waste from orthodontic devices call for immediate action in managing waste sustainably by overcoming distinctive biomedical plastic pollution issues.

### 1.3. Importance of Biodegradation as a Sustainable Solution

Biodegradation has become an attractive and sustainable solution in response to the environmental problems associated with PET accumulation. By following the natural metabolic capabilities of existing microorganisms, this approach utilizes their enzymatic activity to break down complex polymers into environmentally benign products: carbon dioxide, water, and biomass, helping reduce the environmental persistence of plastic waste [10, 33]. While physical recycling can lead to downcycling or is energy intensive and chemical recycling can produce hazardous intermediates during the process, biodegradation provides the ability to completely mineralize PET under ambient conditions consistent with the principles of green chemistry and circular bioeconomy. Synergistic interactions and metabolically complementary enzymatic arsenals in complex communities of diverse bacterial and fungal species, termed microbial consortia, have shown the ability to degrade PET better relative to monocultures [34]. In these consortia, these consortia utilize cooperative metabolic pathways comprising distinct microorganisms that perform sequential or parallel degradation steps to address the metabolic bottlenecks that obstruct PET degradation in single-strain systems [35]. Recently, specific PET-degrading enzymes, especially PETase and MHETase from *I. sakaiensis*, were identified and characterized to identify the molecular mechanisms of PET polymer chains cleavage into monomeric units (TPA and EG), which are then assimilated as carbon sources [36]. Enzymatic depolymerization of PET represents a crucial first step, facilitating further microbial metabolism and mineralization of PET. The combination of biotechnology and sustainable waste management in PET waste from dental plastics, such as orthodontic aligners, is based on an application of microbial consortia–mediated biodegradation. By not only reducing the environmental load of biomedical plastic disposal but also transforming waste into value-added bioproducts or harmless metabolites, this strategy can support future sustainable practices in healthcare. In addition, progress in metabolic engineering, synthetic biology, and process development appears to be propelling the bio-based microbial PET degradation systems toward their potential scalability and practical implementation [11, 37]. Herein, we synthesize current knowledge regarding microbial consortium–mediated PET degradation for biomedical applications. The enzymatic and microbial mechanisms are critically evaluated, biodegradation performance metrics are evaluated, efficacy is characterized in terms of influencing factors, and the environmental implications of the PET waste arising from orthodontic devices are reviewed. This work will identify research gaps and begin to explore emerging biotechnological innovations to inform and inspire future efforts for the development of sustainable bioremediation strategies for PET-based dental plastics.

## 2. PET: Structure and Properties

### 2.1. PET in Orthodontic Aligners

**Figure 2:**
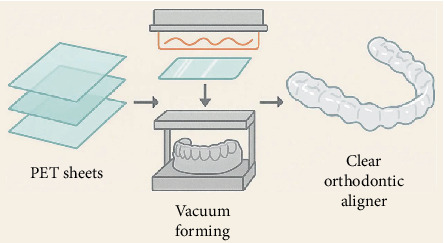
Orthodontic aligners made from PET.

A widely used synthetic polymer, belonging to the polyester group, is PET. Polyesters are synthesized by the polycondensation reaction between TPA and EG, forming a linear thermoplastic polymer with repeating ester functional groups. PET chemistry involves a combination of aromatic terephthalate units linked with an aliphatic EG moiety that makes PET rigid and yet at the same time provides rigidity and flexibility [38, 39]. The desirable mechanical and chemical properties of PET, combined with its transparency, strength, and durability, result from this structural composition. Extensive use of PET in biomedical applications, in large part because of its excellent tensile strength, optical clarity, and resistance to wear, has been made in biomedical devices, for example, orthodontic aligners where aesthetics and biocompatibility are important factors (Figure 2) [21, 40]. Due to patient preference for noninvasive and nearly invisible orthodontic treatments, the demand for PET and its copolymers is also increasing [41]. In terms of its molecular structure, PET is a semicrystalline polymeric material that gives both amorphous and crystalline parts. PET can have its degree of crystallinity ranging from 20% to 60% depending on processing conditions and heat history [42]. Crystalline domains form directly from the ordered packing of polymer chains, promoting tensile strength, stiffness, and chemical resistance of the polymer. Its transparency and impact resistance are due, conversely, to the amorphous regions. The nature of this dual-phase morphology allows PET to trade off between optical clarity and mechanical robustness, both of which are important for the use of PET in biomedical applications, for example, as an orthodontic aligner [43]. Due to the importance of crystallinity in aligner manufacturing, an accurate balance of crystallinity is important, as excessive crystallinity causes decreased transparency, while insufficient crystallinity decreases mechanical properties.

PET's glass transition temperature (Tg) and melting temperature (Tm) define the thermal properties of PET. PET has a Tg of 70°C–80°C, above which temperature the polymer changes from a rigid glassy state to a more flexible rubbery state. That thermal stabilization of the crystalline fraction is indicated by a Tm of 245°C–265°C [44]. The thermal characteristics of PET determine its processing window and end-use performance. PET sheets are dimensionally stable at oral usage since the Tg is above human body temperature [45] and, for example, PET sheets are thermoformed at elevated temperatures to manufacture orthodontic aligners. A second key property of PET is the resultant aromatic polyester backbone that imparts excellent chemical resistance. PET is, in general, resistant to hydrolysis and many solvents under neutral and mildly acidic or basic conditions due to the stability of ester linkages [46]. Nevertheless, hydrolytic cleavage of the ester bonds of the polymer can occur with prolonged exposure to strong alkaline or acidic environments, elevated temperatures, or the action of enzymes resulting in polymer chain scission. PET has inherited this resistance to degradation as the reason for them to persist in the environment and biomedical wastes, making them difficult to recycle and dispose of [47]. The mechanical and chemical properties of PET, coupled with its excellent dimensional stability and substantial biocompatibility, have made it ideal for use with direct intraoral applications such as for clear orthodontic aligners [48]. By engineering the polymer's surface to enhance its wettability and reduce bacterial adhesion, the polymer is hygienically suitable and patient-safe [49]. In addition, PET has optical clarity, so aligners can be almost invisible when worn, a feature that is essential in increasing orthodontic treatment compliance [50]. However, PET is biologically inert and therefore problematic from an environmental perspective. Although relatively heat stable, common environmental microorganisms are unable to break down the strong aromatic ester bonds, resulting in accumulation in landfills and natural habitats [51]. As a result, research has continued into chemical and enzymatic degradation pathways of PET designed to degrade its structure in order to reduce its environmental footprint while preserving the functional benefit in medical devices [52].

### 2.2. Chemical Structure and Physicochemical Properties of PET

PET is a thermoplastic polyester synthesized through the polycondensation reaction between TPA (or its dimethyl ester) and EG. The resulting polymer consists of repeating ester linkages (-COO-) that connect aromatic terephthalate units to aliphatic EG segments [53]. The chemical structure can be represented as repeating units of [−O − CH2 − CH2 − O − CO − C_6_H_4_ − CO−]n[-O-CH_2-CH_2-O-CO-C_6_H_4_-CO-]n[−O − CH2 − CH2 − O − CO − C6H4 − CO−]n.

The semicrystalline nature of PET arises from the combination of a rigid aromatic ring and flexible aliphatic chains. Its mechanical and optical properties vary depending on the variation of the degree of crystallinity, ranging from 20% to 60%, depending on the processing and thermal history [54]. Tensile strength, stiffness, and chemical resistance are generated by crystalline domains of closely packed polymer chains, whereas amorphous regions endure impact and optical clarity [55]. PET has a Tg of 70°C–80°C, separating the rigid and rubbery states, and a Tm of 245°C–265°C, at the melting of the crystalline region [56]. By defining these parameters, the processing window for thermoforming of PET sheets into orthodontic aligners is established, with dimensional stability required in service at body temperature. But strong acids or bases or enzymatic attack can hydrolyze the ester bonds and cause polymer chain scission [57, 58]. Physically, PET is hydrophobic, of high molecular weight, and has, over the past 50 years, been associated with problems for biodegradation due to its low water uptake and poor microbial colonization capabilities. The physicochemical aspects of PET give it high tensile strength, excellent dimensional stability, and optical clarity, leading to the use of transparent medical devices in orthodontic aligners. Wettability and bacterial adhesion are minimized, improving biocompatibility, through surface modifications [59]. The combination of its properties enables widespread adoption of PET in biomedical and packaging industries.

### 2.3. Application of PET in Orthodontic Aligners and Dental Devices

PET possesses unique mechanical properties, optical clarity, and biocompatibility, which have made it an ideal material for manufacturing clear orthodontic aligners and other dental devices. Dentists now have a virtually invisible, removable alternative to traditional metal braces to help with orthodontic alignment that has revolutionized dental treatment and made it more comfortable and a lot easier for the patient to comply [60]. PET's transparent nature allows aesthetically pleasing aligners to maintain a close fit to dental anatomy, allowing effective tooth movement without the need for the wearer to draw attention to them. As a semicrystalline, PET offers the strength and stiffness needed to apply controlled orthodontic forces without deformation in use [61]. The thermoforming of sheets into accurate dental impressions, along with a priori customized fit, is made possible by its thermal properties. Wound healing for PET (Tg ~70°C–80°C) with predictable and consistent outcomes [62] has been enabled by its dimensional stability within the temperature range of the oral cavity. But it is not just limited to aligners; PET is also used in temporary crowns, bridges, and splints because it is durable and easy to fab. In its surface, it can be modified to resist bacterial colonization and prevent oral infections during prolonged wear [63]. Moreover, PET is recyclable and can be reprocessed, making it an environmentally sustainable option for dental material management [13]. Most recently, innovations have focused on PET copolymers and blends to improve mechanical flexibility and mitigate the tensile stress relaxation seen in pure PET within long-term orthodontic environments [64]. Current research also explores combining antimicrobial agents with PET matrices for improved oral hygiene during orthodontic treatment.

## 3. Challenges in PET Degradation due to Its Recalcitrant Nature

PET has very favorable properties for medical and packaging applications but has a chemistry and semicrystalline structure that makes it extremely recalcitrant to natural degradation processes. However, the aromatic ester bonds that comprise the backbone of PET are characterized by high tensile strength and chemical resistance, but they are also rendered low sensitive to hydrolytic or enzymatic cleavage at ambient conditions [65]. PET's hydrophobicity and high molecular weight limit microbial colonization and enzyme access into this polymer, thereby inhibiting biodegradation in soil and aquatic environments [66]. Additionally, the semicrystalline morphology also enhances the degradation resistance, as the crystalline regions restrict water diffusion and enzyme infiltration, while the amorphous regions are easier to get to but represent a smaller fraction of the whole polymer [67]. Surface cracking and embrittlement may be induced by environmental factors, for example, UV radiation and thermal oxidation, slightly increasing susceptibility, but such abiotic processes are slow enough and ineffective for substantial degradation of PET [68]. Traditional waste management methods, for example, incineration and landfills, are polluted, and the last value of material is lost, stressing the necessity for effective recycling or biodegradation technologies [69]. However, microbial degradation by specialized bacteria and fungi producing PETase and MHETase enzymes has been promising, albeit very slow and contingent on optimized environmental conditions [11], and due to the recalcitrance of PET, novel production strategies that fuse enzymatic formations of Marcelo, celery, and microbial consortia and subject the material to redesign in a manner that enhances biodegradability are desirable. Incomplete simultaneous mineralization of organics is a challenge, as is integration into existing waste management systems and scale-up of bioprocesses. Since PET use is increasing in medical devices such as orthodontic aligners [70], these barriers must be overcome to reduce PET's environmental impact.

### 3.1. Environmental Impact of PET Waste From Orthodontic Aligners

**Figure 3:**
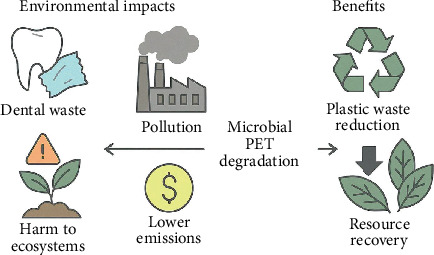
Environmental impact and benefits of microbial PET degradation in dental waste management.

With increasing global demand for clear orthodontic aligners, fabricated predominantly from PET and its copolymers, there has been an increasing generation of biomedical plastic waste associated with high environmental concern. The lifespan of orthodontic aligners is generally 1–2 weeks long; it is easy to imagine that many tons of materials based on PET are sent to the dump every year [1]. Conventional plastics used in packaging enter separate waste flows but typically still flow into landfills and incineration plants, which can have a very detrimental environmental impact [71]. PET is a highly stable aromatic polyester backbone, resulting in physical, chemical, and biological inertness. The stability of this polymer helps keep it around for so long; PET waste will persist in the environment for hundreds of years, with or without active recycling and degradation [47, 72]. Thus, the accumulation of discarded orthodontic aligners contributes to the growing plastic pollution problem that adversely disrupts habitat for terrestrial and aquatic ecosystems and releases microplastics [73]. Persistent organic pollutants and heavy metals, which bioaccumulate in food chains, are also readily adsorbed on fragmented PET, making microplastics derived from fragmented PET a risk to wildlife and human health (Figure 3) [74]. Additionally, existing disposal methods of PET-containing orthodontic wastes only add to environmental problems. However, long-term sequestration occurs as PET reaches the landfill, although there is a risk of leachate generation and contamination of soil. Despite reducing solid waste volume, incineration can release toxic gases such as dioxins and furans, contributing to air pollution and leading to associated health risks [75]. The specialized nature of dental waste and the absence of protocols for standardized recycling employed for other types of PET materials prevent effective diversion of waste in addition to the recovery of resources that otherwise could be utilized. With the carbon footprint created by the manufacture, distribution, and disposal of PET aligners adding to greenhouse gas emissions and contributing to climate change [76], the urgent need for sustainable management strategies for biomedical plastics is highlighted because the cumulative environmental burden of PET waste is presented, developing biodegradable or bio-based alternatives, enhancement of microbial degradation technologies, and circular economy principles within the orthodontic practice [77].

### 3.2. Quantification of PET Waste From Orthodontic Practices

The popularity of clear orthodontic aligners made from PET or its copolymers has dramatically increased the amount of biomedical plastic waste being produced across the world by orthodontic practices. Unlike traditional fixed appliances, PET aligners are replaced frequently, from 1 to 2 weeks, to incrementally change the teeth's position, leading to the disposal of many PET aligners by one patient over one treatment cycle [78]. On average, an orthodontic patient uses between 20 and 40 PET aligners during treatment—that is several hundred grams of plastic waste [1]. Aggregated over millions of patients globally, the cumulative volume of PET waste generated by orthodontic aligners demonstrates significance and is now being measured at dental clinics through surveys and waste audits, in which the plastic components of orthodontic materials, including aligners, are reported to elevate the level of biomedical plastic waste. For instance, a study estimated a single large orthodontic practice to produce 15–20 kg of PET waste annually from aligner disposal alone [79]. This number does not include other PET-based consumables such as retainers and thermoformed splints. For instance, projections indicate that the clear aligner market will grow rapidly, at a compound annual growth rate of over 20% in a few years to come [80], and with that growth will come a rise in PET waste production. As such, quantification efforts have also been directed toward microplastic release during the use and disposal of aligners. PET micro- and nanoparticles that are released intraorally and in the environment after disposal are themselves potential additional sources of environmental and health risk due to mechanical wear and degradation [81]. While these concerns exist, data on the volume and fate of actual orthodontic PET waste are limited; yet, based on inconsistent standards and a lack of centralized collection systems in most regions, quantification of orthodontic PET waste emphasizes the need for the development of new protocols for orthodontic PET waste management, recycling strategies, and biodegradable alternatives to redress this growing reservoir of plastic pollution.

### 3.3. Persistence of PET in the Environment and Associated Risks

PET possesses remarkable chemical stability and physical durability and its persistence in the environmental compartments: soils, freshwater, and marine environments. Aromatic ester linkages in PET make PET very resistant to hydrolysis, oxidation, and microbial degradation and make PET persist on the timescale of decades to centuries in the natural environment [82]. This recalcitrance results in the accumulation of PET debris and microplastics globally in surface waters and sediments and even in remote areas such as the Arctic [83], with environmental persistence of PET from orthodontic aligners and other biomedical waste associated with these microplastics a multifaceted risk. While not all wildlife actually ingest microplastic/mycrobe containing sorbent particles, any time two items get mixed into one, there can be health risks, and this is proved [84]. Additionally, PET microplastics also serve as vectors for toxic chemicals which are problematic additives or absorbed environmental pollutants that bioaccumulate along food chains and which may also enter human consumers [85] or PET microplastics and nanoparticles can adsorb persistent organic pollutants and heavy metals and modify their transport and bioavailability in the environment [86]. Due to their small size, microplastics can be readily taken up by many organisms, from plankton to higher trophic levels, and can result in cellular damage or cause oxidative stress and inflammatory responses [87]. PET particles may change soil structure and affect aeration and water retention, potentially causing issues for plant growth and soil microbial communities [88], but an important concern in terrestrial systems is the oral ingestion of microplastics from dental devices, a relatively new concern, but human health impacts are not yet well understood and require further study. Also complicating remediation of PET is its long residence time, as it does not readily mineralize and thus can persist as a long-term pollutant. However, these environmental risks underscore the urgent need to sustainably manage PET waste and invent biodegradable substitutes to reduce the ecological footprint of PET.

## 4. Current Disposal Practices and Their Limitations

Disposal of PET waste from orthodontic aligners represents an enormous challenge for biomedical waste management systems. Landfilling, incineration, and less frequently mechanical recycling are common disposal methods, but each has serious limitations. Despite that, landfilling remains the major method for disposing of these wastes, particularly in those areas that do not possess specialized medical waste treatment infrastructure. PET waste deposited at a landfill will be sequestered, but environmental persistence can lead to decades of survival as the polymer remains intact [89]. While PET waste can be destroyed by incineration to reduce volume and potentially recover energy, poor control can result in dioxins, furans, and particulate matter emissions [90]. Incineration emissions pose an environmental and health concern, especially in densely populated areas where incinerators operate without modern scrubbing technologies for the emission of noxious gases from the combustion of PET orthodontic waste [75]. Mechanical recycling of orthodontic waste is constrained by contamination, the absence of segregation, and thermal degradation of the polymer quality during repeated processing [91]. The recovery and recycling of biomedical plastics are often complicated by mixing them in streams of other infectious waste. Furthermore, the limited volume and unique nature of orthodontic PET waste eliminate economic motivations for dedicated recycling systems, and chemical recycling techniques such as glycolysis or hydrolysis present the potential for depolymerization of PET to yield monomers that can be repolymerized; however, these require high costs and technical complexity [92]. Additionally, there is no standardization of protocols for PET orthodontic material collection and disposal, which are further contributing to environmental leakage and landfill buildup, and the current disposal practices are inadequate to limit the overall environmental impact of the PET waste from the orthodontic aligners. Innovative, sustainable waste management strategies, including alternatives like biodegradable ones, microbial degradation technologies, and improved regulatory approaches to recycling, are in dire need for support of recycling and minimization of ecological footprint.

### 4.1. Microbial Degradation of PET: An Overview

**Figure 4:**
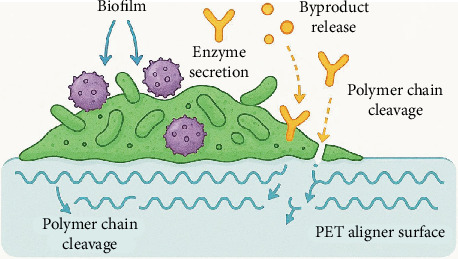
Mechanism of microbial degradation on PET surface.

Synthetic polyester PET has good durability and is chemically resistant and is consequently widely used for packaging and biomedical applications. But recalcitrant it is and that makes life hellish on the environment because it lasts for decades in ecosystems. The biotechnological solution to the plastic pollution problem is microbial degradation of PET employing microorganisms capable of hydrolyzing ester bonds in the polymer backbone (Figure 4) [93]. PET hydrolases such as PETase and MHETase are key enzyme catalysts of PET depolymerization to its monomer, TPA and EG [94]. In 2016, the discovery of *I. sakaiensis* was a milestone in the diversification; this bacterium has both PETase and MHETase, allowing efficient PET degradation under laboratory conditions. Since then, various homologous enzymes and additional PET-degrading microbes have been identified in diverse environments [65]; microbial consortia consisting of bacteria and fungi with complementary enzymatic capabilities can degrade PET better than each individual strain. In turn, the sequential enzymatic steps of these consortia together could synergistically degrade PET, increase hydrolysis rates, and also expand environmental adaptability [95]. Temperature, pH, PET crystallinity, and surface properties are, in fact, all factors that strongly influence biodegradation efficiency. Despite progress, however, microbial PET degradation still has challenges, including slow degradation rates, incomplete mineralization, and scalability of bioprocesses [69]. For example, amorphous PET regions degrade faster than highly crystalline domains with better enzyme accessibility. The use of protein engineering to engineer PET hydrolases has improved enzyme stability and activity and has improved their feasibility for industrial applications [96]. In addition, the immobilization techniques and the bioreactor designs are being optimized for better degradation under controlled conditions. In general, microbial degradation of PET constitutes a promising and sustainable alternative to mechanical or chemical recycling. Breaking down the remaining limitations requires continued research in order to reach large-scale biodegradation large enough to reduce PET pollution in the environment appreciably and scale toward a circular bioeconomy.

### 4.2. Types of Microorganisms Known for PET Degradation

A number of microorganisms—bacteria, fungi, and actinomycetes—mediate the microbial degradation of PET employing a complex array of enzymatic capabilities leading to partial or even total breakdown of this recalcitrant polymer. PET degraders are the most extensively studied bacteria, several of which are capable of hydrolyzing PET into the monomeric components. Although the discovery of *I. sakaiensis* from a PET-contaminated environment was a groundbreaking one, enabling the isolation of a bacterium specializing in the depolymerization of PET by a specific enzymatic system composed of PETase and MHETase enzymes catalyzed the depolymerization of PET into TPA and EG. Beyond *I. sakaiensis*, the bacterial genera *Pseudomonas*, *Bacillus*, *Thermobifida*, and *Cutinimonas* have been reported to degrade PET or PET-like polyesters by secreting extracellular hydrolases [97]. Fungi serve important roles in PET degradation, particularly through the secretion of a diverse set of extracellular enzymes capable of degrading more complex polymers, and such bacteria reside in environments rich in organic polymers such as compost, landfill soils, and wastewater treatment plants. Ligninolytic enzymes of white-rot fungi such as *Phanerochaete chrysosporium* and *Trametes versicolor*, namely, laccases and peroxidases, have been well recognized to oxidatively degrade synthetic polymers (PET) [14]. Further, species of *Aspergillus, Fusarium*, and *Penicillium* isolated from plastic-polluted environments have been shown to catalyze PET degradation, often in consortia with bacteria [16]. Generally, fungal degradation runs slower than bacterial degradation; however, it can accelerate under particular circumstances. Filamentous Gram-positive bacteria, actinomycetes, are recognized for their potent enzymatic toolkit for the degradation of recalcitrant compounds and bridge the characteristics of fungi and bacteria. Cutinases and polyesterases capable of hydrolyzing PET substrates have been reported for the species of *Thermobifida* and *Streptomyces* [19]. The filamentous growth of actinomycetes enables them to colonize the solid surfaces of plastic materials, making enzymatic access possible. Their thermophilic variants facilitate PET degradation at elevated temperatures, which is advantageous for industrial bioprocesses; however, through synergistic metabolic activities in microbial consortia, these microorganisms often enhance the rates of PET degradation beyond those that can be achieved with a single strain. Consortia such as these have complementary enzyme expression, improved colonization, and adaptation to environmental stresses [98].

### 4.3. Microorganisms Known for PET Degradation: Bacteria, Fungi, and Actinomycetes

PET is an important durable polymeric material currently employed in packaging, textiles, and biomedical devices such as orthodontic aligners. Widespread exposure of PET in the environment has stimulated intense research into microbial pathways for degradation, and bacteria, fungi, and actinomycetes have been identified as potential key players in degrading this polymer, enzymatically breaking down into monomers. Cultured bacteria, especially *I. sakaiensis*, identified, have been the most studied PET degraders. *Ceramides* are then dispatched to normally aerated muscles, where they taxi away from the injury and away from the otherwise previously flightless neighbors that have had their wings torn off. Where flies sense an edge condition, the fly's behavior is altered, with previous flightless neighbors regaining their flight capability [99]. Other bacterial genera, however, including *Thermobifida*, *Pseudomonas*, *Bacillus*, *Rhodococcus*, and *Cutinimonas*, have been shown to degrade PET using extracellular polyesterases and cutinases [65, 82]. Of special note is PET hydrolysis conducted by thermostable enzymes secreted by thermophilic bacteria such as *T. fusca*, which are active at elevated temperatures and thus greatly accelerate PET hydrolysis [19]; fungi are major participants in PET degradation due to their powerful oxidative enzymes. *P. chrysosporium* and *T. versicolor*, white-rot fungi, produce laccases and peroxidases that will oxidize aromatic rings of PET, weakening its structure and making it hydrolytic cleavage susceptible [14]. While many bacterial species have also been found to be able to hydrolyze PET-like polyesters, including *P. putida*, there are also other fungal species known to hydrolyze PET-like polyesters such as *Aspergillus*, *Fusarium*, and *Penicillium*, typically in synergy with bacteria [100]. Biodegradation typically occurs more slowly by fungi than by bacteria, but fungi have a vital role in the complex microbe community by changing the chemical surface of PET and making it more accessible. Actinomycetes, filamentous bacteria with fungal-like characteristics, have been found to contain very diverse extracellular enzymes, including cutinases and polyester hydrolases. Among these species, *T. fusca*, *S. viridis*, *Micromonospora*, and *Nocardiopsis* are known for their hydrolysis of PETs in particularly well-composed environments, with organic matter [97]. PET can be degraded by actinomycetes at high temperatures; in addition, filamentous growth of actinomycetes assists in colonizing plastic surfaces, which increases degradation rates. Next to individual strains, microbial consortia containing bacteria, fungi, and actinomycetes have shown more PET-degrading activity than monocultures due to their having robust enzymes under industrial conditions that are attractive for biotechnological application (Table 2). Sequential and complementary enzyme actions are facilitated by synergistic interactions, allowing oxidative enzymes from fungi [101] to oxidize PET surfaces, increasing the accessibility of bacterial hydrolase to PET. The concept of “adaptation” to environmental stresses is explored as one of the reasons these consortia obtain more efficient degradation and support their potential for PET bioremediation.

## 5. Enzymatic Mechanisms Involved in PET Breakdown

**Figure 5:**
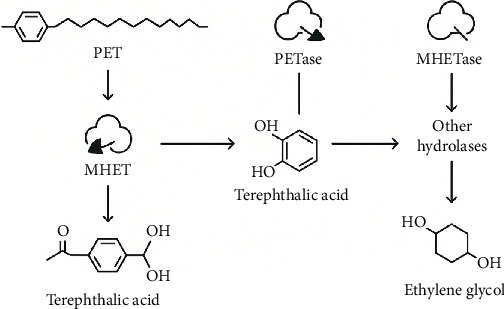
Enzymatic pathway of PET degradation.

Specialized enzymes that catalyze the hydrolysis of PET ester bonds drive its fundamental microbial degradation, whereby the polymer is hydrolyzed into its monomeric constituents, TPA and EG. The most widely studied of these enzymes are PETase and MHETase, comprising the most efficient two-step enzymatic system to date, found in *I. sakaiensis* [102]. Structurally, PETase is a serine hydrolase, with a cleft for binding the substrate, optimized for accommodating the PET polymeric chain to target for cleavage. Since PETase activity, MHETase hydrolyzes mono (2-hydroxyethyl) terephthalate (MHET) into TPA and EG, finishing the depolymerization pathway; protein engineering efforts further improved its catalytic efficiency and thermostability, broadening its potential application (Figure 5) [52]. This enzyme shows high specificity for MHET to avoid accumulation of toxic intermediates and enhance downstream microbial metabolism [36]. Along with the enzymes described above, the enzymes of *I. sakaiensis* together make a surprisingly effective PET degrader. For example, thermostable *F. cutinases* and polyesterases from *Fusarium* spp. drill into the surface of the amorphous regions of PET [103]. Degradation of PET is typically enzymatic and is generally said to proceed through adsorption to the PET surface, polymer chain cleavage, and desorption of the products; recent advances suggest the critical role of enzyme synergy and structural adaptations that improve access to PET's semicrystalline regions. Enzyme performance is further enhanced by immobilization and optimization of reaction conditions to make the enzymes useful for phytoremediation and recycling applications.

## 6. Microbial Consortia Versus Single Microorganism Approaches

### 6.1. Concept and Advantages of Microbial Consortia in Biodegradation

**Figure 6:**
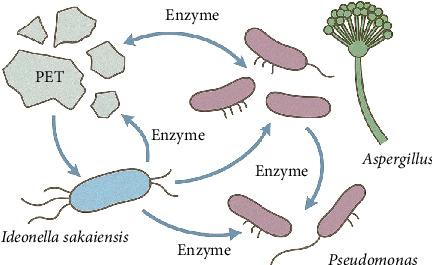
Microbial consortium involved in PET degradation.

Microbial consortia are structured communities of two or more microbial species that interact synergistically or complementarily for functions typically impossible or too inefficient to be accomplished by a single microorganism (Figure 6). Consortia take advantage of the differing metabolic capabilities and enzyme repertoires of diverse microbes to degrade complex pollutants such as synthetic polymers more readily. Compared to monocultures, microbial consortia exhibit ecological robustness, metabolic versatility, and functional redundancy and can tolerate diverse and dynamic environmental conditions [104]. In this regard, microbial consortia are able to divide complex biochemical pathways among different species. Depolymerization of polymer by one organism creates intermediate compounds to be finished off by the further action of other members, which reduces feedback inhibition and achieves complete mineralization [105]. Division of labor results in a lower metabolic burden for individual microbes and in ineffective utilization of substrates. Consortia also possess enhanced tolerance to environmental stresses such as pH shifts, temperature fluctuations, and toxic intermediates, although members provide synergistic protection and resource sharing [9]. However, a single microorganism can produce only one or two relevant enzymes, whereas consortia can express a complete ensemble of enzymes conventionally used to speed up the process of breakdown and increase degradation rates [106]. In addition, microbial consortia can promote biofilm formation on plastic surfaces to increase localized enzyme concentration and substrate contact time, which enhances biodegradation [107]. As such, microbial consortia provide a systems-based and ecologically meaningful way to address bioremediation, improving efficiency, resilience, and scalability of PET degradation and other environmental interventions.

### 6.2. Synergistic Interactions Enhancing PET Degradation

Synergistic interactions between microbial communities are essential for enhancing the degradation of PET and other persistent polymers. Metabolic cooperation, enzymatic complementarity, and environmental modification are three forms of these interactions that allow the breakdown of substrates through greater efficiency. Metabolic cooperation is a form of interaction between microbial populations involving the indirect cooperation in degrading a substrate, in which intermediate degradation products generated by one population serve as substrates for another. For example, it is known that polymer hydrolysis proceeds from the polymer to MHET or to TPA, which, in turn, some microbes may accumulate to inhibitory levels. The continuous polymer degradation is prevented by accumulation or feedback inhibition by the presence of other species that can metabolize these intermediates [108]. The sequential metabolism also increases the overall mineralization rate at the expense of breakdown products. Another important mechanism by which microorganisms obtain complementarity in enzymatic processing is enzymatic complementarity, in which different microorganisms produce distinct enzymes that act on various bonds and structural features of PET. One microbe might secrete PETase directed at ester bonds while another secretes oxidative enzymes such as laccases or peroxidases to modify aromatic rings, thus making the substrate more susceptible to hydrolysis [18]. The combination of this multienzyme system broadens the range of degradable PET fractions, most notably the crystalline domains that are typically resistant to degradation; environmental modification includes microenvironmental modification factors such as pH, oxygen levels, and biofilm matrix composition dictated by members of these consortia. Synergistic extracellular polymeric substances (EPSs) that are produced by a number of bacteria and fungi facilitate the formation of biofilms on the PET surface, concentrating enzymes and providing better contact with the substrate [109]. Alternatively, others are able to locally modify the pH or secrete surfactants that increase the PET wettability, thereby promoting enzyme adsorption and enzyme activity [110].

### 6.3. Examples of Consortia Used for PET or Similar Polymer Degradation

It has been shown in several studies that microbial consortia degrade PET and related polymers better than single strains. For instance, a consortium of three organisms, *I. sakaiensis*, *P. putida*, and *Bacillus subtilis*, was able to enhance PET degradation with a combination of synergistic hydrolytic and oxidative activities under composting conditions [16, 111]. In marine environments, *T. versicolor* and *Pseudomonas* spp. mixed fungal–bacterial consortia were effective in degrading PET films by integrating the fungus laccase-driven oxidative process with bacterial hydrolysis [112]. Once again, thermophilic consortia dominated by *T. fusca* and *Streptomyces* species catalyzed accelerated PET hydrolysis with thermostable enzymes and may provide promising industrial-scale applications [19], while landfill-derived microbial communities harboring *Streptomyces*, *Bacillus*, and fungal genera have been identified to efficiently degrade PET and polyurethane plastics compared to isolated strains, demonstrating the ecological relevance of consortia for polymer biodegradation in natural environments [93].

### 6.4. Microbial Consortium–Mediated PET Degradation in Orthodontic Aligners

Due to its favorable mechanical strength, optical clarity, and biocompatibility, PET, which forms the core polymeric material in many clear orthodontic aligners, is particularly attractive. The chemical stability and semicrystalline structure of PET confer remarkable resistance to the natural degradation of PET-based biomedical wastes, for example, discarded orthodontic aligners in the environment [113]. This waste will persist and persist and is ecologically risky stuff, requiring inventive biodegradation. Microbial consortia have been developed as a potentially superior solution because their additional enzymatic activities and synergies can accelerate the efficiency of PET degradation beyond that of single microorganisms. Microbial consortia consist of complex bacterial, fungal, and actinomycete communities whose interlocking metabolism and supporting enzymatic complements lead to a full breakdown of PET. Unlike monocultures that could deliver a single specific enzymatic function, consortia provide a variety of enzymatic tools in the larval arsenal (PETases, cutinases, lipases, esterases or laccases, and peroxidases). The synergistic action of these enzymes, which attack PET ester bonds, aromatic rings, and polymer surface properties, is essential in allowing for efficient PET depolymerization in PET orthodontic aligners [52, 114]. To use one example, PETase and MHETase are secreted by bacteria like *I. sakaiensis* and allow them to harvest the monomers EG and TPA from PET. At the same time, aromatic intermediates are metabolized by complementary bacteria such as *Pseudomonas* spp. and thereby are not allowed to accumulate in toxic amounts. Simultaneously, fungal members such as *T. versicolor* release laccases to petrologically modify PET surfaces, thereby enhancing hydrolytic susceptibility to the bacterial enzyme [15]. *T. cutinases* from actinomycetes such as *T. fusca* increase degradation rates under industrially relevant conditions and contribute thermostable cutinases [115].

Additionally, microbial consortia are also effective in the formation of biofilms on the hydrophobic surfaces of the orthodontic aligners. The biofilm matrix concentrates degradative enzymes in a concentrated environment and increases substrate contact time, resulting in increased hydrolysis kinetics [116]. Furthermore, consortia exhibit cooperative metabolism, which confers resilience to environmental stresses and thus are apt for application in a waste treatment facility or composting operation aimed at treating biomedical plastics, as recent studies have shown microbial consortia specifically adapted to degrade PET in orthodontic aligners. Through optimized conditions, consortia of *Ideonella*, *Bacillus*, and *Aspergillus* species were shown to dramatically increase the degradation rates of PET films derived from aligner material, with these consortia achieving substantial weight loss and surface erosion in less than a week. Besides that, additional pretreatment techniques such as UV exposure or mechanical fragmentation can enhance microbial accessibility and hence quicken biodegradation processes [117]. While significant advances have been made toward microbial-based consortiums, there are still challenges to scaling microbial consortium–based PET degradation to be used at the large scale to manage such orthodontic waste. Further research is required to control community composition, maintain enzymatic activity under variable environmental conditions, and integrate degradation processes into existing biomedical waste frameworks. Metagenomics, synthetic biology, and enzyme engineering advances promise to enable tailoring consortia that carry optimized PET degradation pathways for orthodontic applications.

### 6.5. Specific Studies on Microbial Consortia Targeting PET in Orthodontic Aligners

Clear orthodontic aligners made primarily of PET or PET-based copolymer have shown a substantial increase in their use worldwide because of their aesthetic and functional advantages. Nevertheless, the recalcitrance of PET makes the resultant biomedical waste an environmental concern. Due to the collective enzymatic diversity and synergistic capabilities, microbial consortia have been investigated as promising agents for the biodegradation of these polymeric materials. Several recent studies have been devoted to identifying and characterizing microbial consortia capable of PET degradation from orthodontic aligners. Holding under bottled laboratory conditions, [18] also reported a defined consortium including *I. sakaiensis*, *Bacillus subtilis*, and *Aspergillus niger*, which degraded the PET films obtained from discarded orthodontic aligners. The establishment of the consortium's enzyme profile was attained, which was able to break down more PET than monocultures, and a large surface erosion was observed (more than 60%) after 30 days of bioreactor operation. Similarly, Salinas et al. [17] have isolated a marine-derived fungus–bacteria consortium consisting of *T. versicolor* and *P. putida*, with evidence for the degradation of PET substrates similar to aligners through laccase and PETase synergistic activities.

Specific research efforts on microbial consortia–driven degradation of orthodontic aligner PET have increased over the past few years, with a number of studies leading the wave showing positive outcomes. The most striking study was where a consortium that included *I. sakaiensis*, *P. putida*, and *B. subtilis* was synthesized. This community was chosen with caution after the important enzymatic functions, such as the breaking of PET polymers, TPA derivative metabolisms, and biofilm formation on the surfaces of the polymers, were deemed complementary [118]. Over 60% mass reduction was attained by the consortium upon a 30-day bioreactor experiment using PET films that were made using discarded orthodontic aligners [119]. Surface erosion was determined by SEM, and monomeric degradation products were released, and the products were confirmed through FTIR and HPLC, thus showing both physical and chemical breakdown of the aligner material [120, 121].

A promising development was provided that generated a marine-derived consortium of *T. versicolor* and *P. putida* [122]. In that arrangement, the fungal component began to oxidize PET on the surface by secreting laccase, greatly improving the availability of bacterial PETases [123]. Their experiment system thermoformed with PET-G sheets made of orthodontic aligner-grade polymers had a 45% degradation level after 3 weeks. In this experiment, oxidative pretreatment was revealed to be significant in improving the biodegradability of high-crystallinity PET surfaces to microbial degrading activities [124]. Additional experimental methods have included thermophilic consortia, with species in them including *T. fusca* and *Streptomyces* spp. exhibiting polyesterase and cutinase activity at higher temperatures. These types of consortia have been model tested in rotating disc bioreactors run under composting-mimic conditions to give an expandable format of installing microbial degradation into biomedical waste systems. In such instances, the biodegrading process was much slower in aligners that had not been UV pretreated, which is yet another example of the importance of treatment of surfaces [120].

With these improvements, literature has continued to say that the orthodontic PET has a high resistance to wear compared to general beverage PET. Discrepancy is credited to variances in polymer crystallinity, additives copolymerized to ensure flexibility and clarity, and multilayered structures employed as an impediment to enzyme penetration. In addition, dental materials usually possess some kind of plasticizers, antibacterial agents, or even color to which microbial proliferation activities may be sensitive, thereby making degradation kinetics quite cumbersome [125, 126]. The future trend in the area will look into employing genome engineering tools to boost the activity of targeted enzymes, developing bioaugmentation approaches working with consortia on immobilized carriers, and real-time tracking of biodegradation processes using omics-based instruments [127, 128]. Moreover, microbial PET degradation incorporated into decentralized treatment units in dental clinics and aligner manufacturing facilities in general might provide a more viable alternative to incineration and landfill systems. Such developments will not only be promising in terms of environmental protection but also in sync with the rest of the globe in terms of attaining a circular and sustainable biomedical economy in relation to orthodontic practice.

## 7. Experimental Setups and Analytical Techniques

**Figure 7:**
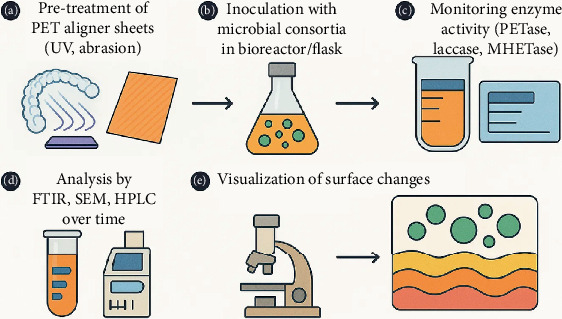
Experimental setup for microbial degradation of orthodontic PET aligners.

Evaluation of the breakdown of PET from orthodontic aligners by microbial consortia includes keeping samples of the material under defined or natural types of microbes at fixed conditions. It is common to keep microbial and enzyme activities high by running experiments at 30°C–50°C and at pH values of 7–8 [129]. Usually, commercial aligner sheets cut to appropriate dimensions serve as PET substrates. These are normally given a UV treatment or roughened mechanically to improve their surface and make enzymes better able to access them. In order to observe biodegradation closely, intermediate sample collection intervals are applied during the incubation period, which lasts several days or weeks. Rates of biodegradation are accurately measured using different laboratory methods. By using gravimetric methods, it is possible to estimate and monitor how PET mass is reduced over different periods, which gives important data about bulk polymer degradation. However, due to a lack of sensitivity to small molecular changes, extra characterization techniques are often necessary. Using scanning electron microscopy (SEM), the group signaled different kinds of changes such as pitting, cracks, loss of material, and biofilm creation that showed PET was being broken down by physical and enzymatic processes [130]. In addition, both Fourier-transform infrared spectroscopy (FTIR) and x-ray diffraction (XRD) give useful information about the changes in chemicals and structures. FTIR can spot ester bond splitting and the creation of functional groups, revealing polymer damage, and XRD highlights lower crystallinity, meaning the polymer chains have been disrupted and microbial enzymes can reach them more easily [131]. HPLC and GC-MS detect degradation ingredients like MHET, TPA, and EG to uncover the degradation mechanisms and confirm the success of bioenzymatic methods. In addition, doing assays for PETase, MHETase, laccase, and cutinase gives a clear picture of how well the consortium can degrade various materials. Although experiments in the laboratory showed that about 10%–35% of the material decomposed within 30–60 days, more adjustments are needed to reach standards important for industry and economics (Figure 7).

## 8. Factors Influencing PET Biodegradation

The way bacteria degrade PET aligners depends on the mix of microbes, the conditions in the environment, and intrinsic features of the aligners. Synergy among bacteria and fungi with different functions and enzymatic reactions encourages complete polymer degradation [33]. Factors in the environment affecting biodegradation mainly depend on temperature, the level of acidity, and whether there is enough oxygen. Both enzyme efficiency and the number of microbes increase when temperatures range from 30°C to 50°C and pH levels are near or slightly above neutral. Moreover, when conditions are aerobic, the enzymes required to break down PET (depolymerize) function more effectively than under anaerobic conditions [132].

Both the crystallinity and surface features of the substrate greatly determine how quickly biodegradation will occur. Orthodontic aligner PET usually demonstrates good crystallinity and hydrophobicity, which help it in the clinic but harm its ability to break down. Crystalline materials block the access of enzymes to the polymer because they limit the free movement of molecular chains, and surfaces that are not water-loving prevent microbes from sticking and forming a biofilm needed for steady enzyme work [69]. Treating the surface of a material by UV irradiation or abrasion raises its roughness and adds hydrophilicity, making it easier for microbes to adhere and improving enzymatic activity [133]. It is also necessary to consider that microorganisms could be challenged by other compounds used to make aligner devices, making the problem of degradation even more difficult. Tuning both the substrate and environment is important to improving PET decomposition and making these technologies practical for use at large scales.

## 9. Challenges and Biotechnological Approaches

Several difficult problems remain in efficiently biodegrading the PET in orthodontic aligners. Slow deterioration, reduced access to enzymes by the highly ordered structure, and harmful buildup of intermediates and complex compounds in commercially used aligners result in the main challenges [36, 134]. Moreover, problems with keeping microbial communities and enzymes reliable at a large scale are barriers preventing this technology from being widely used. To address these problems, biotechnology is now using enzyme engineering, synthetic biology, and bioprocess improvement. Thanks to protein engineering methods like directed evolution and computational design, the activity, heat stability, and what they can process are now greatly improved in enzymes like PETase and MHETase [135]. The approach of synthetic biology makes it easier to build microbial communities that use optimized pathways and cooperate, which improves the final results of degradation [136]. Meanwhile, using wild-type catalysts in bioreactor designs stops fluctuations in these enzymes and helps preserve the community, allowing biodegradation to happen nonstop under control. Merged studies in microbial ecology, enzymes, materials, and bioprocess engineering are necessary to find effective and economical methods for breaking down orthodontic PET waste.

## 10. Conclusion

The microbial group used to break down PET-based orthodontic aligners points to an environmentally friendly way to handle orthodontic waste. From my findings, I learned that boosting the effectiveness of biodegradation depends on shaping the microbial community, adjusting growth conditions, and adjusting PET-type substrates. Looking at degradation via gravimetric, SEM, FTIR, XRD, and chromatographic approaches gives a clear picture of changes in the PET due to enzymes and microbes. Still, challenges like increased crystallinity of polymers, issues related to water repellency on their surfaces, the buildup of inhibitory intermediates, additives causing microbial inhibition, and limitations on scaling up are still present, pushing the need for fresh biotechnological approaches. Protein engineering, synthetic biology, surface preparation strategies, and advanced bioprocess engineering are essential to dealing with these issues. More studies are needed to create strong groups of microbes and designed enzymes that better break down PET in real-life environments and factories, helping to move toward safe and efficient use of aligners.

## Figures and Tables

**Table 1:** Microorganisms and enzymes involved in PET degradation.

**Microorganism**	**Enzymes**	**Optimal temp (°C)**	**pH range**	**PET type**	**Key findings**	**Reference**
*I. sakaiensis*	PETase, MHETase	30–37	7–8	Film	Complete depolymerization to TPA and EG	[11]
*T. versicolor*	Laccase, MnP	28–32	4–6	Aligner	Oxidizes aromatic rings	[14, 15]
*P. putida*	Esterase, lipase	30–37	6.5–7.5	Film	Assimilates PET monomers	[16, 17]
*B. subtilis*	Hydrolases	30–40	6.5–8	Aligner	Enhances biofilm matrix	[12, 18]
*T. fusca*	Cutinase	50–60	8–9	Crystalline PET	Effective at high temperatures	[19]

**Table 2:** PET degradation using microbial consortia on orthodontic materials.

**Organisms used**	**Substrate type**	**Setup**	**Duration**	**Degradation (%)**	**Analytical tools**	**Reference**
*I. sakaiensis*, *P. putida*	PET film (aligner-grade)	Bioreactor	30 days	~60%	SEM, FTIR, HPLC	[67]
*T. versicolor*, *P. putida*	PET-G thermoformed sheet	Static culture	21 days	~45%	FTIR, SEM	[70]
*T. fusca*, *Streptomyces* spp.	Crystalline PET film	Rotating bioreactor	30 days	~52%	TGA, SEM	[72]
*A. niger*, *P. fluorescens*	PET-G sheet (dental)	Shake flask	25 days	~48%	GC-MS, FTIR	[73]
*B. subtilis*, *R. oryzae*	PET film	Anaerobic digester	40 days	~35%	TOC, SEM	[74]

## Data Availability

The data that support the findings of this study are available from the corresponding author upon reasonable request.
